# Glycolic acid and ultrasonic activation: Effects on smear layer removal, dentin penetration, dentin structure and bond strength of the root dentin filling material

**DOI:** 10.4317/jced.61215

**Published:** 2024-10-01

**Authors:** Matheus Albino Souza, Karolina Frick Bischoff, Rafaela Ricci, Luiza Frick Bischoff, Eduarda Reuter, Nathalia da Silva Gomes, Mariana Gabriela Hofstetter, Eduardo Winck dos Santos, Theodoro Weissheimer, Marcus Vinícius Reis Só, Ricardo Abreu da Rosa, José Antonio Poli de Figueiredo, Huriel Scartazzini Palhano, Yuri Dal Bello

**Affiliations:** 1 School of Dentistry, University of Passo Fundo, Passo Fundo, RS, Brazil; 2 School of Dentistry, Federal University of Rio Grande do Sul, Porto Alegre, RS, Brazil

## Abstract

**Background:**

Glycolic acid (GA) has been tested as enamel and dentin etchant, and final irrigant for smear layer removal. This study evaluated the effects of GA and ultrasonic activation (US) on smear layer removal, dentin penetration, dentin structure and bond strength of the root dentin filling material.

**Material and Methods:**

The root canals of 210 teeth were distributed into four test groups: smear layer removal (N=50), dentin penetration (N=50), dentin structure (N=10) and bond strength (N=100). In all tests, specimens were subdivided into five groups, according to the final irrigation protocols: G1:distilled water(DW)+US; G2:17% EDTA; G3:17% GA; G4:17% EDTA+US; G5:17% GA+US. Scanning electronic microscopy, confocal laser scanning microscopy, transmission electronic microscopy and the push-out test were performed to evaluate smear layer removal, dentin penetration, dentin structure and bond strength, respectively. Statistical analysis was performed for each evaluation (α=5%).

**Results:**

Groups 4 (EDTA+US) and 5 (GA+US) were the most effective on smear layer removal, with no statistical differences from each other (*p*>0.05). The maximum penetration depth into dentinal tubules was observed for group 5(GA+US), being statistically different from all other groups (p*p*<0.05). Group 5(GA+US) showed the largest collagen dispersion area, being statistically different from groups 1(DW+US), 2(EDTA) and 3(GA) (p*p*<0.05), and similar to group 4(EDTA+US) (*p*>0.05). The highest BS values for filling and restorative material were observed for all experimental groups, being statistically different from control group (p*p*<0.05), with higher predominance of cohesive failure.

**Conclusions:**

The association of GA and US promotes better smear layer removal, dentin penetration and collagen dispersion, with no influence on bond strength of the root dentin filling/restorative material.

** Key words:**EDTA, final irrigation, glycolic acid, ultrasonic activation.

## Introduction

The smear layer (SL) is formed during the chemo-mechanical preparation of root canals. It is defined as an amorphous structure containing organic and inorganic compounds, including dentin debris, odontoblastic fragments, microorganisms and necrotic tissue (1). SL adheres to root canal walls and obliterates dentinal tubules, compromising the diffusion of intracanal medication (2), infiltration of endodontic sealers (3) and adhesion of filling materials (4). Moreover, SL increases the possibility of reinfection (5). Thus, SL removal is recommended after chemo-mechanical preparation.

Irrigant solutions with ability to perform adequate dentin penetration are necessary for SL removal. Moreover, the ultrasonic activation (US) of these solutions is recommended to improve their action and penetration into the root dentin. Then, better cleaning and root canal disinfection, collagen dispersion and adequate adhesion of the filling material to root canal walls could be obtained (5). At the same time, it is known that the use of conventional irrigant solutions induces dentin erosion (6) and modification of the dentin structure (7), compromising dental fracture resistance. Thus, new strategies have been searched, preserving the root dentin structure at the same time of promoting SL removal.

Ethylenediaminetetraacetic acid (EDTA) is the main chelating agent on endodontics, with ability to remove SL (8). However, it has reduced action on the apical third (9), it is more cytotoxic (10) and induces significant changes in the dentin structure (11). It also causes indirect effects on cell metabolism and inflammatory regulation (12). Therefore, some alternatives for final irrigation protocol (FIP) should be identified, promoting effective dentin penetration and SL removal, at the same time, preserving the dentin structure and improving the adhesion of root canal filling/restorative materials.

Glycolic acid (GA) belongs to the alpha hydroxyl acid group that also includes citric acid, being originally used for skin treatment (13). Recently, GA has been used as surface enamel and dentin etchant, presenting satisfactory results (14). Moreover, it has ability to remove SL and low cytotoxicity (10), with no ability to induce modification in the mechanical properties of the root dentin (15). In your turn, the US is recognized as an important resource, enhancing the properties of irrigant solutions applied to the root canal (16). Although previous findings have shown that association of GA with US does not interfere in some mechanical properties of the root dentin (15), there are no studies in literature showing the effects of this association for SL removal, dentin penetration, dentin structure and the bond strength (BS) of root canal filling/restorative materials.

The aim of this study was to evaluate the effects of GA and US on SL removal, dentin penetration, dentin structure and BS of root dentin filling/restorative materials. The hypotheses were that this association (i) promotes significant SL removal (ii) and root dentin penetration, (iii) preserves the dentin structure and (iv) increases the BS of root dentin filling/restorative materials .

## Material and Methods

The present study was approved by the Institutional Review Board (protocol 4.436.756).

-Smear layer removal evaluation 

Fifty single-rooted extracted human teeth with single and straight root canal were obtained from the local Biobank. Dental crowns were sectioned with diamond disc so that all roots remained with 15 mm in length. The working length (WL) was determined by inserting a 10 K-file (Dentsply-Sirona, York, PA, USA) until its tip was visible at the apical foramen. WL was defined by subtracting 1 mm from this point. The apex was externally covered with silicone impression material (Zetaplus; Zhermack, Polesine Badia, Italy) to prevent solution leakage.

Roots were enlarged to WL using ProTaper rotary system (Dentsply-Sirona), from S1 to F3 file, and irrigated with 2 mL of distilled water (DW) using NaviTip 30-G tip (Ultradent Products Inc, South Jordan, UT, USA) and 5-mL syringe, renewing the irrigant solution on each instrument change. Each root canal was irrigated with 5 mL of DW and dried with absorbent paper points (Tanari, Manacapuru, AM, Brazil).

Grooves were created on the buccal and lingual root surfaces with a diamond disc, avoiding intrusion into the root canal space. Half of each tooth was scanned under SEM at low-vacuum mode (VEGA LM 3, Tescan, Libušinatř, Kohoutovice, Czech Republic), with 1 kV accelerating voltage. Three marks were made on root external surfaces using a fine-tip pen, dividing them into equal thirds (cervical, middle and apical) along their longitudinal axes. These marks served as a guide for preparing three grooves in the root canal wall. Grooves were made using a diamond disc. A size-11 scalpel blade created an additional 3-mm-long mark perpendicular to axial grooves. Debris from markings were cleared using water-assisted air jets.

Specimens were incubated at 37°C for 48 hours and placed in vacuum desiccator for another 48 hours. Then, they were examined using low-vacuum SEM (VEGA LM 3, Tescan, Libušinatř. Kohoutovice, Czech Republic). The most distinct area (fully covered by SL and easily visible) of cross-shaped marks in each root third was selected. An image was captured at 300x magnification with the area’s edge aligned with the mark’s limits. Another image was taken at 1000x magnification without altering the sample position. Six images were acquired per specimen before FIP testing , serving to assess the initial condition of root canal walls after chemo-mechanical preparation.

Tooth halves were reassembled and stabilized by filling cleavage grooves with light-cured composite resin (Natural Shade,DFL,Taquara,RJ,Brazil). Roots were placed in heavy condensation silicone impression material to prevent solution leakage.

Fifty teeth were randomly divided into five groups (n=10) based on FIP: G1:DW+US; G2:17% EDTA; G3:17% GA; G4:17% EDTA+US; G5:GA+US. A compounding pharmacy prepared all test solutions. In groups without US, the solution was injected into root canals with 5-mL syringe and 30-G needle until reaching the canal entrance, remaining for 1 minute. In groups with US, the solution was applied, followed by US (NacPlus, Adiel, Ribeirão Preto, SP, Brazil). The stainless-steel E1-irrisonic endodontic tip (Helse Ultrasonic, Santa Rosa de Viterbo, SP, Brazil) was inserted 1 mm shorter than the WL and activated for 1 minute at power of 20%. Finally, all groups underwent 5 mL DW irrigation and drying with aspiration cannula.

**Figure 1:**
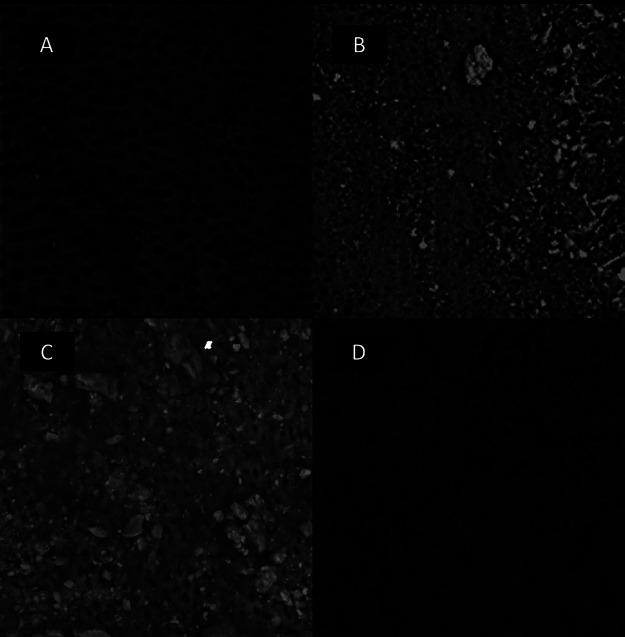
SEM images (x1000) demonstrating the scoring system used to analyze the effectiveness of each group in the smear layer removal. (A) Score 1; (B) Score 2; (C) Score 3; (D) Score 4.

Roots were separated into 2 halves, resulting in 20 halves per group, which were dehydrated in ethanol, mounted on aluminum stubs, coated with gold palladium and examined via SEM in high-vacuum mode (10–20 kV/5.0-mm working distance) (VEGA LM 3, Tescan, Libušinatř. Kohoutovice, Czech Republic). New images from the same preselected areas were captured using a previously reported protocol (17). Subsequently, two blinded and calibrated examiners analyzed the final images taken after FIP using a scoring system adapted from previous study (18): score 1- opened dentinal tubules without debris; score 2 - opened dentinal tubules with debris covering <50% of the area; score 3 - opened dentinal tubules with debris covering >50% of the area; score 4 - dentinal tubules covered by debris in 100% of the examined area (Fig. 1). The Kappa coefficient test indicated good agreement between examiners (k=0.886). Data were analyzed using Kruskal-Wallis and Mann-Whitney U tests for intergroup comparisons, and Wilcoxon and Friedman tests for intragroup comparisons (α=5%).

-Dentin penetration evaluation 

Fifty single-rooted extracted human teeth with the same characteristics were prepared as previously described. After chemo-mechanical preparation, the 50 samples were randomly divided into the same five groups (n=10), as previously detailed. To enable the observation of the irrigant solution penetration into dentinal tubules, irrigants were prepared by a compounding pharmacy and 0.1% fluorescent rhodamine B by weight was added.

Following FIP, root canals were irrigated with 5 mL of DW and dried using aspiration cannula. Subsequently, specimens were fixed on acrylic plates and transversally sectioned at 5 and 10 mm from the apex, using a cutting machine (Isomet 1000;Buehler;Hong Kong,China) under refrigeration at 325 rpm, which resulted in three slices per root. The slice surfaces were standardized to 500 µm thickness by polishing with 1200, 2400, and 4000 silicon carbide abrasive papers (Struers,Westlake,OH,USA).

**Figure 2:**
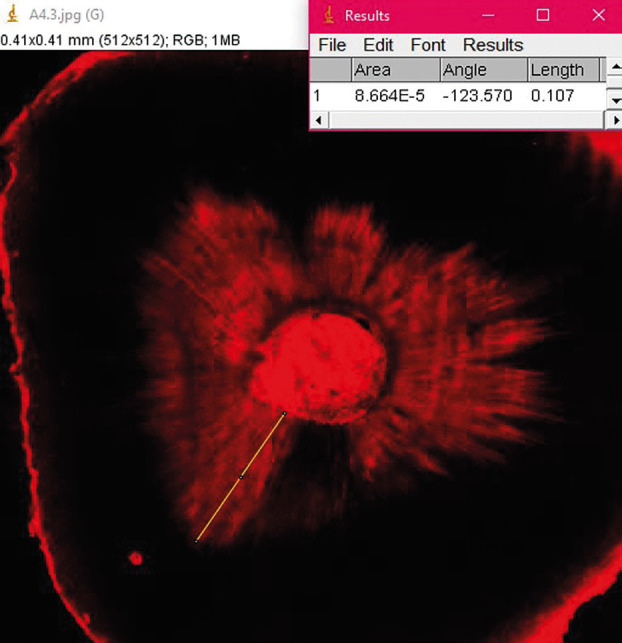
CLSM image illustrating the maximum penetration measurement (in µm) of the tested irrigation protocol in the depth of dentinal tubules.

Samples were examined using a confocal laser scanning microscope (CLSM) (Zeiss LSM-Pascal; Carl Zeiss, Goëttingen, Germany) in epifluorescence mode. The images of areas filled by FIP were acquired at absorption wavelengths and rhodamine B emission wavelengths of 540 nm and 590 nm, respectively. A depth of 10 μm below the surface was analyzed at 500× magnification for each root canal third. These images were imported into the Image J software (National Institutes of Health, MD, USA) to measure the maximum FIP penetration (in µm) for each third (Fig. 2). The average penetration value (in µm) for each sample was calculated based on these three measurements. Two previously blinded and calibrated examiners conducted measurements.

Data were submitted to One-way ANOVA test, followed by Tukey’s post-hoc test for intergroup comparisons (α=5%).

-Dentin structure evaluation 

Ten bovine incisor teeth obtained from animals slaughtered for commercial purposes were transversely sectioned below the cementum-enamel junction, producing 15 mm-long root segments. Two buccal and lingual longitudinal grooves, along with two grooves 5mm and 10mm from the apex encircling the root, were created using a diamond disc. Manual K-type files (Dentsply-Sirona) performed the instrumentation, enlarging the root canal up to a 50 file, with 5 mL DW rinse for every file change. Roots were randomly divided into 5 groups (n=2), following previous procedures.

Roots were divided into two halves, generating four halves per group. Encircling grooves were cleaved, producing four samples from each root canal third. Samples from cervical and apical thirds were discarded, while those from the middle third were evaluated by transmission electron microscopy (TEM), as described in previous study (11). Samples were submitted to decalcification, fixation, dehydration and embedding in silicone molds with resin, followed by storage at 60°C for 72 hours. Ultramicrotomy yielded 100 nm thick sections along the long axis of dentinal tubules, which were treated with 2% Uranyl Acetate and Lead Citrate for contrast. TEM (FEI, Thermofisher Scientific, Hillsboro, Oregon, USA) was used for examination at power of 200 kV.

Each group had eight images captured at 8,900x magnification. Qualitative collagen analysis was performed by a blinded senior examiner, being classified as intact (INT), dispersed (DIS) or altered (ALT).

Quantitative analysis used the Image J software. RGB color adjustment was applied and three areas (dentinal tubule entrances) were selected using the Rotated Rectangle function. The image was duplicated, color-adjusted for binarization and analyzed for collagen presence/absence. The software saved results for areas with and without collagen. This process was repeated in three areas of the original image surrounding the dentinal tubule entrance. Results for the three rectangles were exported to Excel, differentiating total area, white area (without collagen) and black area (with collagen), obtaining the average for each image.

Qualitative data were not submitted to statistical analysis. Quantitative data were submitted to One-way ANOVA, followed by the LSD test for multiple comparisons, determining black (collagen) and white (collagen dispersion) area percentages for each group (α=5%).

-Bond strength evaluation 

One hundred single-rooted extracted human teeth with the same characteristics were prepared as previously described. Fifty teeth were used to assess the BS of the filling material and fifty teeth for BS of the restorative material, which were randomly divided into five groups (n=10), as previously described.

In the first assessment, roots were filled with gutta-percha and AH Plus sealer (Dentsply-Maillefer), by lateral condensation technique. In the second evaluation, glass fiber posts (GFP) No.1 (FGM, Joinvile, SC, Brazil) were cleaned with 35% phosphoric acid for 30s, rinsed for 30s and air-dried. Silane application (3M ESPE) for 1 min was followed by Single-Bond (3M ESPE) adhesive application and 40s of light polymerization with halogen light source (600 mV/cm2) (Optilux, Demetron Res. Corp, Danbury, CT, USA). The root canal was not etched. Rely-X U200 self-adhesive cement was inserted using Centryx syringe and Acudosse needle (DFL, Rio de Janeiro, RJ, Brazil). GFP was covered with cement and placed into the root canal at 10 mm level, being held under digital pressure for 20s. Excess cement was removed and polymerization for 30s on each face was performed.

All specimens were stored at 37 degrees in 95% humidity for 21 days to set the filling/restorative material. Roots were transversely sectioned from the root canal entrance into 1-mm-thick discs using metallographic cutter (Isomet 1000, Buehler, Hong Kong, China) with diamond disc under cooling at speed of 350 rpm. The first disc was discarded and the next five discs were selected from each sample, totaling 50 specimens per group in each evaluation. Each disc underwent the push-out test in mechanical testing machine (Emic DL 2000, São José dos Pinhais, PR, Brazil) at speed of 1 mm/min, until material displacement. Care was taken to ensure that the punch tip contacted the material over the widest area. The force required for material displacement was recorded in newton (N) and calculated in megapascal (MPa).

BS in MPa was determined using the formula δ=F/A, where F is the force (N) and A is the area. To calculate the area, the equation A=2πr x h was applied, with π being the constant value of 3.14, r being the radius of the intra-radicular space and h the height (mm). Failure modes were categorized under optical microscopy (Zeiss, São Paulo, SP, Brazil) at 50x magnification: 1-adhesive: between dentin and the filling/restorative material, absence of material on dentine walls; 2-cohesive: failure of the filling/restorative material, presence of material on dentine walls; 3-mixed: both failures. Data were submitted to One-way ANOVA followed by the Tukey post hoc test, and failure mode distribution was assessed using the chi-square test (α=5%).

## Results

The mean and standard deviation values of SL removal, dentin penetration, collagen area (black area–%) and dispersion area (white area–%) for each group are presented in  Table 1.

All images obtained at low vacuum after root canal preparation revealed significant presence of SL. The intergroup analysis revealed that groups 4(EDTA+US) and 5(GA+US) were the most effective in SL removal, with no statistical differences from each other (*p*>0.05). The intragroup analysis revealed no significant differences between groups 1(DW+US) and 3(GA) (*p*>0.05), whereas SL removal was significantly more deficient in the apical third in the other groups (*p*<0.05).

**Figure 3:**
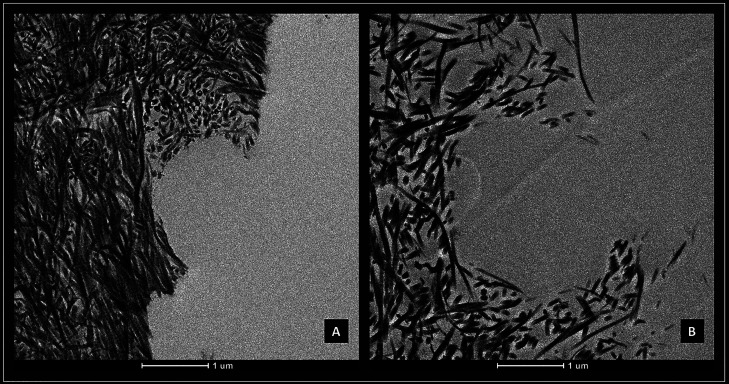
TEM images illustrating (A) intact and (B) dispersed collagen.

Group 5(GA+US) exhibited the maximum penetration depth into dentinal tubules, significantly differing from all other groups (*p*<0.05). Subsequently, group 4(EDTA+US) demonstrated higher dentin penetration compared to groups 1(DW+US), 2(EDTA), and 3(GA) (*p*<0.05). TEM qualitative analysis revealed INTACT status for group 1(DW+US) and DISPERSED status for all other groups, as illustrated in Figure 3. The quantitative analysis showed that group 5(GA+US) showed the largest collagen dispersion area, being statistically different from groups 1(DW+US), 2(EDTA) and 3(GA) (*p*<0.05), and similar to group 4(EDTA+US) (*p*>0.05).

 Table 2 and  Table 3 present the mean and standard deviation values for the BS of filling/restorative materials, and the failure pattern percentage in each group. In both evaluations, all experimental groups exhibited higher BS values, being statistically different from the control group (*p*<0.05), with no significant differences from each other (*p*<0.05). The chi-square test found no statistically significant differences in the failure pattern among groups, with higher predominance of cohesive failure in both evaluations (*p*>0.05).

## Discussion

GA was the alternative irrigant solution chosen for this study, as it presents recognized ability to remove SL (10) and low surface tension (19), which can be favorable for its penetration into the root dentin and collagen dispersion. However, the impact of US over GA in variables tested in the present study is not well elucidated in literature. Regarding the contact time with root canals, FIP was performed for 1 minute in this study. Considering that SL can be effectively removed in a short irrigation time, in addition to the fact that excessive irrigation time can lead to erosion and crack formation in the root dentin (20), contact time of 1 minute was adopted.

The control group (DW+US) consisted of ultrasonic activation of distilled water. According to the findings of previous studies, there was no statistically significant difference when the DW and DW+US protocols were compared in the removal of smear layer promoted by endodontic instrumentation or in the removal of chemical smear layer formed by the photosensitizer of photodynamic therapy (21,22). Considering these results and the significant difficulty in obtaining extracted human teeth, as well as the knowledge that inert solutions do not have the ability to promote smear layer removal (4), the isolated use of DW as control group was not performed in the present study.

SEM is usually performed to evaluate SL removal, based on the amount of SL and opened dentinal tubules (6). To ensure reliable evaluation, a predefined area was first analyzed with low-vacuum SEM before the use of FIP, and the same area was examined after FIP by high-vacuum SEM to quantify the SL removal efficacy. The association of rhodamine B to tested irrigant solutions was based on a previous study, providing adequate conditions to visualize their penetration into the root dentin by CLSM (23). In addition, the maximum penetration depth (μm) into root dentinal tubules was measured for each group, with basis in previous studies that evaluated the penetration of irrigating solutions, antimicrobial agents and endodontic cements into the depth of dentinal tubules (20,24,25). It is an easier and faster method that allows this way of evaluation. TEM corresponds to an effective resource for assessing the collagen morphology and structural defects of the root dentin, acting in qualitative and quantitative analyses (6). Finally, the push-out test represents a consolidated method to evaluate BS of root canal wall filling materials, being comparable with the clinical reality, which induces effective power for material displacement and less stress at the bond interface (26). Therefore, these tests were adopted to evaluate the variables of the present study.

According to the present study, US improved the ability of irrigant solutions to remove SL in all root canal thirds, confirming the first hypothesis. Ultrasonic vibration promotes hydrodynamic turbulence, where temperature increase and hydrostatic pressure are induced, producing cavitation and bubbles that collide against the root canal walls (27). Then, better SL removal was reached in this study. The present results also revealed high difficulty for SL removal in the apical third, even when US was performed. Considering that oscillation amplitude is greater on the ultrasonic tip, any interference may significantly affect the apical portion (28), in addition to the fact that irrigation with conventional needles leads to lower ability to deliver the irrigant solution to the apical third (9).

Some bacterial species present ability to colonize the root canal and penetrate the dentinal tubules, such as Enterococcus faecalis (29). In these areas, the infection has progression, leading to failure of the endodontic therapy. In this scenario, dentin penetration by irrigant solutions is essential, whether to provide decontamination or create a path for the penetration of antimicrobial agents in these anatomical complexities. The maximum penetration depth was observed for group 5(GA+US), confirming the second hypothesis of the present study. This can be explained by the low pKa, low molecular weight and low surface tension of GA, providing greater action on the mineral surface and penetration into dentinal tubules (19), as well as by the association of US, which makes the irrigating solution to reach deeper regions of the root dentin. Moreover, GA presents higher antimicrobial activity when compared to EDTA (30), being an additional resource used in the decontamination process, at the same time contributing to remove SL.

Bovine teeth ensured standardized collagen patterns for TEM evaluation (6). The experimental groups treated with US showed the largest collagen dispersion area in this study, confirming the third hypothesis. Wagner *et al*. (6) revealed displacement of the collagen surface and thinning of fibrils, as well as extensive erosion of peritubular and intertubular dentin when EDTA was used. However, it was observed through qualitative analysis, associating the use of EDTA with NaOCl. The present study performed quantitative analysis, being possible to calculate the dentin collagen dispersion area , in addition to qualitative analysis. The preservation and adequate dentin collagen dispersion is essential for the penetration of adhesive systems into the root dentin. The adhesive materials are then bonded to root canal walls, contributing to the durability of restorations with cemented intracanal posts (31).

According to previous studies, the use of US over the tested final irrigants improved the BS of the filling/restorative material (32,33). These findings are not in accordance with the present results, rejecting the fourth hypothesis. The epoxy-resin-based endodontic sealer, AH Plus, presents cohesion between molecules and satisfactory flowability, increasing the filling material displacement resistance (33). In your turn, the presence of acidic monomers in the composition of Rely-X U200 self-adhesive resin cement demineralizes the root dentin, resulting in micromechanical retention Reaction between acid monomers and hydroxyapatite of the dentin substrate was also observed, resulting in chemical retention (34). The association between mechanical and chemical retention helps to explain the effect of BS on the root dentin in this study, as well as the higher incidence of cohesive failure. Moreover, discs were obtained from the cervical and beginning of the middle third, where large volume of irrigating solution is delivered, even without the aid of US. This may also have contributed to the lack of significant differences between groups.

The small size of GA molecules and their acidic pH contribute to an effective demineralization of the root dentin, resulting in effective SL removal from root canal walls (10), which is the main proposal of a chelating solution. Moreover, GA presents some advantages when compared to EDTA (10,15,19,30). At the same time, the literature demonstrates several benefits of US for cleaning and decontamination in endodontic therapy. Thus, this study suggested the use of GA with US as a promising alternative for FIP, contributing for cleaning in endodontic therapy and preservation of the dentin structure for further restorative procedures.

Under the study limitations, it was possible to conclude that the association between GA and US promotes better SL removal, dentin penetration and collagen dispersion, with no influence on the BS of the root dentin filling/restorative material.

## Figures and Tables

**Table 1:** Mean and standard deviation of smear layer removal scores, maximum penetration depth (μm) into root dentinal tubules, collagen area (black area – %) and dispersion area (white area – %) for each group.

Group	Smear layer Cervical third (Scores)	Smear layer Middle third (Scores)	Smear Layer Apical third (Scores)	Maximum penetration depth (μm)	Collagen Black area (%)	Dispersion White area (%)
1. DW+US	4.00± 0.00^A,a^	4.00±0.00^A,a^	4.00±0.00^A,a^	92.45±14.04^A^	63.00±2.08^A^	22.33±2.40^A^
2. EDTA	2.60±0.11^B,a^	2.62±0.07^B,a^	2.83±0.09^B,b^	75.91±13.42^A^	40.33±4.70^B,C^	16.33±6.06^A^
3. GA	2.71±0.14^B,a^	2.57±0.13^B,a^	2.85±0.19^B,a^	96.22±11.79^A^	48.00±4.50^B^	36.33±4.37^B^
4. EDTA+US	2.10±0.09^C,a^	2.20±0.06^C,a^	2.45±0.14^C,b^	119.07±8.22^B^	44.33±3.92^B,C^	37.00±8.00^B,C^
5. GA+US	1.95±0.18^C,a^	2.10±0.12^C,a^	2.50±0.09^C,b^	146.08±12.28^C^	32.33±8.41^C^	50.66±5.84^C^

* Different capital letters indicate significant differences between groups. Different small letters indicate significant differences between root thirds (*p*< 0.05) ** DW=distilled water; US=ultrasonic activation; EDTA=Ethylenediaminetetraacetic acid; GA=glycolic acid.

**Table 2:** Mean (standard deviation) of bond strength of filling material to root canal dentin (MPa) and percentage of pattern of failure (%) of tested final irrigation protocols.

Group	n	Push Out Bond Strength	Failure mode		
			Adhesive	Mixed	Cohesive
1. DW + US ^a^	50	1.76 (1.17)	28.00	12.00	60.00
2. EDTA ^b^	50	7.91 (4.27)	28.00	12.00	60.00
3. GA ^b^	50	12.21 (5.93)	40.00	8.00	52.00
4. EDTA + US ^b^	50	15.55 (7.50)	8.00	10.00	82.00
5. GA + US ^b^	50	20.31 (8.78)	8.00	12.00	80.00

* Different superscript lowercase letters indicate, in the column, statistically significant differences (*p*< 0.05). ** DW, distilled water; US, ultrasonic activation; EDTA, Ethylenediaminetetraacetic acid; GA, glycolic acid.

**Table 3:** Mean (standard deviation) of bond strength of restorative material to root canal dentin (MPa) and percentage of pattern of failure (%) of tested final irrigation protocols.

Group	n	Push Out Bond Strength	Failure mode		
			Adhesive	Mixed	Cohesive
1. DW + US ^a^	50	3.15 (1.89)	12.00	32.00	56.00
2. EDTA ^b^	50	10.04 (4.16)	18.00	24.00	58.00
3. GA ^b^	50	14.69 (5.28)	30.00	20.00	50.00
4. EDTA + US ^b^	50	14.74 (5.97)	26.00	24.00	50.00
5. GA + US ^b^	50	18.05 (6.62)	20.00	26.00	54.00

* Different superscript lowercase letters indicate, in the column, statistically significant differences (*p*< 0.05). ** DW, distilled water; US, ultrasonic activation; EDTA, Ethylenediaminetetraacetic acid; GA, glycolic acid.

## Data Availability

The datasets used and/or analyzed during the current study are available from the corresponding author.
